# Implant success!!!…..simplified

**DOI:** 10.4103/0972-124X.51891

**Published:** 2009

**Authors:** Kaushal K. Luthra

**Affiliations:** *Professor and Head of the Department, Department of Periodontology and Implantology, M.M.College of Dental Sciences and Research, Mullana, Ambala, India*

**Keywords:** Aesthetic wax-up, implant, radiographic template

## Abstract

The endeavor towards life-like restoration has helped nurture new vistas in the art and science of implant dentistry. The protocol of “restoration-driven implant placement” ensures that the implant is an apical extension of the ideal future restoration and not the opposite. Meticulous pre-implant evaluation of soft and hard tissues, diagnostic cast and use of aesthetic wax-up and radiographic template combined with surgical template can simplify the intricate roadmap for appropriate implant treatment.

By applying the harmony of artistic skill, scientific knowledge and clinical expertise, we can simply master the outstanding implant success in requisites of aesthetics, phonetics and function.

## INTRODUCTION

Patients with missing teeth in the appearance zone generally do not directly ask for implants. They would like to have their teeth replaced in the most elegant and long-lasting way possible. Implant surgery aims at providing long lasting anchorage in the best possible position for a functionally and aesthetically optimal restoration.

The success of an implant-supported restoration relies on the proper implant body placement. Successful implant placement requires accurate angulation and position to achieve functionally satisfying results. This includes criteria such as maximum preservation of sound tooth structure, avoidance of removable prosthesis, and minimal risk during surgical and healing phases as well as cost-effectiveness.

Meticulous preoperative planning and three dimensional implant integration protocols are of immense importance for successful implants. These include the principles of case selection, case evaluation, proper planning, pre-operative preparation and optimal implant placement and implant specified definitive restoration.

## ESSENTIALS OF SUCCESS IN IMPLANT RESTORATION

- Clinical examination
- Pre-clinical evaluation
- Aesthetic wax-up
- Radiographic template
- Surgical template
- Three dimensional implant placement
- Implant specified restoration

### Clinical Examination

#### Periodontal biotype

There are two different periodontal biotypes that have been described in relation to the morphology of the interdental papilla and the osseous architecture:

1. The thin scalloped periodontium: The thin scalloped periodontium is characterized by a thin and scalloped osseous housing of the tooth and by a thin gingival tissue with long interdental papillae. It has the tendency to develop soft tissue recession in response to trauma or periodontal infection.
2. The thick flat periodontium: The thick and flat periodontium is characterized by a thick osseous structure and flat morphology, thick gingival tissue and short and wide papillae. The thick periodontal biotype is relatively resistant to surgical trauma and recession and the presence of periodontal infection most often leads to pocket formation.

### Osseous topography

The health status and quality and quantity of bone must be assessed in future implant sites using clinical and radiographic parameters. The clinical assessment includes inspection and palpation of the edentulous area in consideration to decide any persistent pathology or defect and volume of the bone available for implant placement.

The bone volume available should allow the implant placement in pristine bone. The bone morphology has to be visualized using osseous sounding to avoid a dehiscence or fenestration during the drilling phase.

### Occlusion

The clinical examination must include a judgment of the interarch, interdental space for future prosthetic construction. When occluding, a minimum interarch distance must be at least 5 mm to harbor the implant supported restoration. The smallest interdental space that can be accepted without damaging the periodontal support of neighbouring teeth is around 7 mm, if implants of about 4mm diameter are to be used.

### Radiographs

Radiographic examination of peri-implant tissues is an essential diagnostic requirement for the assessment of success and stability as well as failure of dental implants. The assessment of bone support in endosseous dental implants is fundamental to the clinical utility of implants for restoration for function.

Radiographs are a critical tool for the assessment of bony architecture, and radiographs are used at each of the three phases of implant treatment, evaluation and maintenance.

Intraoral films are utilized in pre-surgical planning of implant treatment, intra-operatively, and for longitudinal assessment. Periapical radiographs are used to assess limited areas or individual implant sites. The periapical films have minimal distortion if well angulated and are suitable for evaluation of bone height.

Occlusal films are used to assess the buccal to lingual width of the edentulous ridge area during the pre-surgical planning phase.

## RESTORATION DRIVEN IMPLANT PLACEMENT PROTOCOL

### Diagnostic casts and working models

The value of diagnostic casts or study models is critical, especially in oral implantology. Securing dental cast of the patient and mounting these casts on the articulator will provide a great deal of information about the existing oral conditions that may not be apparent during the oral examination. The diagnostic mounting offers the opportunity to design optimal occlusal contact and to determine the need for additional restorative care. The diagnostic cast enables the dentist to evaluate several prosthodontic criteria in the absence of the patient as discussed below:

- Occlusal centric relation position, including premature occlusal contacts
- Edentulous ridge relationships to adjacent teeth and opposing arches
- Position of potential natural abutments including inclination, rotation, extrusion, spacing, parallelism, and esthetic considerations

Selection of the implant design for a patient can initially be made from the diagnostic mounting. Once, the implant has been selected, the choice of the surgical procedure can be considered.

During treatment, planning mounted casts are essential for diagnosis and fabrication of implant positioning devices. The diagnostic wax-up on the working models provides a vision of the emergence and position of the definitive implant supported restoration.

With the vision of definitive restoration in hand, a comprehensive examination will provide the team members with information that can help maximize aesthetic outcomes.

## RIDGE MAPPING WITH IMPRESSION TRACING AND BONE SOUNDING

### Impression tracing method

An Elastomeric impression material is recommended to make a dual arch impression of the site. The impression mass is then removed and the site length measured. The impression mass is then bisected facio-lingually with a laboratory Bard Parker knife to give two arch forms of the proposed site. The gingival, interocclusal spaces measured will be added to the gingival thickness to give the bone opposing dentition distance. The arch form is then traced on paper in the patient record which is in fact the gingival contour of the site.

### Bone sounding

About four to six lines are marked on the cut surface of the putty index, perpendicular to the mucosa, approximately 7 mm apart on labial surface, crestal region and on the palatal surface. The edentulous area is then anesthetized with local infiltration. The endodontic reamer with rubber stopper is used to measure the thickness of the soft tissue along the lines marked on index. The measurements are recorded.

### Ridge mapping

So, each recorded measurement is noted as a point under arch tracing. The points are then connected to give another form which is an approximation of the underlying bone contour.

### Evaluation of buccolingual width

The buccolingual bone dimensions can be now measured on the tracing to give the surgeon information as to appropriate implant sizing diameter. Then, the tracing so obtained can be superimposed with the transparent radiographic guide provided by the manufacturer to evaluate the dimensions and direction of implant placement. A diameter implant of a ridge, too large or too thin, may produce a dehiscence. The 5 mm level is the depth to which the implant should be placed to avoid subsequent exposure of the implant thread due to resorption of the thin bone.

### Aesthetic wax-up

The clinician must envision the completed prosthesis to observe the desired position of teeth and soft tissues with the help of diagnostic wax-up.

### Radiographic template

After satisfactory mouth testing, the study prosthesis is duplicated in acrylic resin and then serves as a scanning template. A linear path is drilled through the main axis of the resin tooth. A gutta -percha cone is inserted to relate the ideal prosthetic axis so that it is clearly visible on the X-ray radiograph.

### Surgical template

To establish a logical continuity between the planned restoration and surgical phases, it is essential to use a transfer device. An integral part of pretreatment planning includes designing a surgical guide that will be used to direct implant placement buccolingually, mesiodistally, and apicocoronally.

The surgical guide template is fabricated by the restoring dentist after the pre surgical restorative appointments, once the final prosthesis, optional abutment(s) number and location, occlusal scheme, and implant angulation have been determined.

However, long axis of the implant should lie in a more palatal position that corresponds to a deeper housing of the implant head, about 4-5 mm from the buccal soft tissues of the adjacent teeth. The position of the head permits enough transition room to restore the emergence profile of the crown.

The surgical template communicates the actual implant position to the surgical site. The surgical template dictates to the surgeon the implant body placement that offers the best combinations of

- support for the repetitive forces of occlusion
- aesthetics
- hygiene requirements.

The template can be used, not only in critical anatomical situations but also in placing the implant in an ideal position on bone, because it eliminates possible manual placement errors and matches planning according to prosthetic requirements. A precise template is useful, particularly in cases in which positioning is critical, because of anatomical conditions.
